# The American Dream: Is Immigration Associated with Life Satisfaction for Latinos of Mexican Descent?

**DOI:** 10.3390/healthcare11182495

**Published:** 2023-09-08

**Authors:** Vickie M. Mays, Rosina Becerra, Susan D. Cochran

**Affiliations:** 1 Department of Health Policy and Management, UCLA Fielding School of Public Health, Los Angeles, CA 90095, USA; 2 Department of Psychology, University of California, Los Angeles (UCLA), Los Angeles, CA 90095, USA; 3 UCLA BRITE Center for Science, Research and Policy, University of California, Los Angeles (UCLA), Los Angeles, CA 90095, USA; 4 Department of Social Welfare, UCLA Luskin School of Public Affairs, Los Angeles, CA 90095, USA; 5 Department of Epidemiology, UCLA Fielding School of Public Health, Los Angeles, CA 90095, USA; 6 Department of Statistics, University of California, Los Angeles (UCLA), Los Angeles, CA 90095, USA

**Keywords:** nativity, social support, life satisfaction, quality of life, Mexican, immigrant, well-being, psychological distress

## Abstract

The Latino population is one of the largest immigrant groups in the United States, with the majority being of Mexican descent. Whether immigrating to the US is positive for the well-being of Mexican immigrants and future generations is an important question. We examined how nativity status and quality of life indicators relate to life satisfaction among foreign-born and US-born Mexican descent Latinos living in California. Participants (N = 893) were from the California Quality of Life Survey, a population-based mental health survey of the California population. Multiple regressions examining sociodemographic and indicators of life satisfaction found higher life satisfaction among the foreign-born compared to US-born: (US-born first generation: Wald F = 18.70, *p* < 0.001; US-born second generation and higher: Wald F = 12.09, *p* < 0.001), females (Wald F = 7.05, *p* < 0.01), and individuals reporting more social support (Wald F = 40.20, *p* < 0.001), absence of frequent distress (Wald F = 41.46, *p* < 0.001), and better physical health (Wald F = 15.28, *p* < 0.001). Life satisfaction was lower for US-born Mexicans than for Mexican immigrants. Research, interventions, and policies are needed for mental health equity that address this lack of well-being in US-born Mexican Latinos.

## 1. Introduction

For many Latinos, particularly those of Mexican descent, there is great optimism about life in the United States (US) both for themselves and their children [1,2]. Latinos remain one of the fastest-growing racial/ethnic minority groups in the US, with more Latinos residing in California than in any other state [3,4]. Approximately one-quarter of all Latinos in the US live in California, giving California a great investment in enhancing their well-being and life satisfaction. The majority of Latinos in California are of Mexican origin [4]. Many migrated here for opportunity resulting in a significant growth of the Mexican-origin population from the 1980′s to 2000 [5]. The close proximity of low- and high-wage economies resulted in a flow of Mexican immigrants in search of economic opportunity and a better life [6]. Approximately half of Mexicans believe that life will be better in the US and about one-third say they would move to the US if they had the opportunity to do so [2].

The term “American dream” is often associated with the aspirations of immigrant groups [7]. The American dream refers to the belief that achievement and success are determined by an individual’s efforts and hard work, and not restricted by class structures such as socioeconomic status or race/ethnicity [7]. In the context of immigration, the American dream is closely tied to beliefs about the potential for prosperity of families and future generations. Despite all of the focus on immigration as a path to fulfilling dreams of opportunity, we know little about whether immigrating to the US is positive for the emotional well-being of Mexican immigrants and future generations. The US has experienced an economic recession, increased difficulty for immigrants to become citizens, and an increased anti-immigrant sentiment [8,9,10]. These changes in the economic, social, and political context of the US may impact experiences and beliefs about the achievability of the American dream and well-being among Latino immigrants and their children. 

There are few representative studies whose goal is to compare life satisfaction among Mexican immigrants and US-born Mexican Americans living in the US to examine whether the optimism about the dreams and opportunities reported by foreign-born immigrants is perceived in later generations born or reared in different circumstances. The present study examined life satisfaction among individuals of Mexican origin living in California and focused on the role of nativity to examine whether those who came to the US as immigrants or those born here of immigrant or US parents remain optimistic about the American dream and still carry the sense of life satisfaction of those who immigrated here directly from Mexico. The focus on nativity is important because of the demographic shift of the Mexican-origin population in the US. After decades of immigration largely driving Mexican-origin population growth in the US, beginning in 2000, native births became the leading driver of Mexican population growth [11], however, with immigration being such a contentious issue, currently there is a decline. Furthermore, sociodemographic characteristics (e.g., income, education, age), as well as psychosocial experiences, important factors for life satisfaction, have been shown to differ for Mexican immigrants and Mexican Americans born in the US [11].

### 1.1. Life Satisfaction

Life satisfaction is considered a critical component of well-being and quality of life, both of which were added as major target areas to Healthy People 2020 [12]. When an individual’s life circumstances are similar to his or her standards, life satisfaction is considered to be high. This standard referent is subjective, varying from one individual to the next [13]. Life satisfaction is an important construct because it is considered critical to having a healthy lifestyle and has been associated with health outcomes, even mortality [14]. Research on life satisfaction has identified the important role of sociodemographic characteristics, for example, marital status, income, and education have all been shown to influence life satisfaction [15]. Research on the role of nativity status, an important sociodemographic characteristic in particular among those of Mexican origin in the US, however, is limited. The present study takes a bottom-up model approach when conceptualizing life satisfaction [16]. The bottom-up approach considers satisfaction with life to be the result of one’s experiences. Satisfaction in different domains, for example, with one’s social support system, health, and education, all help determine overall life satisfaction. This approach is in contrast to the top-down model which considers satisfaction with life to be a personality trait that is relatively stable over time and is not influenced by changes in life conditions [16]. 

Studies that have examined life satisfaction among Latinos remain limited and findings are inconsistent, with some studies showing that Latinos in the US report lower levels of life satisfaction as compared to other racial/ethnic groups, while others find that Latinos report higher levels of life satisfaction, e.g., [17,18,19]. In a 2019 study examining disparities in the “happy paradox” of Latinos, it was found that older Latino immigrant adults experienced the highest levels of life satisfaction compared to Blacks, other races, and Whites [20]. Fewer studies have focused on life satisfaction among individuals of Mexican origin and many of these studies focus on subpopulations, such as older adults [21,22] and youth [23]. Among general Mexican-origin populations living in the US, in a study of 1138 foreign-born adults, overall, Mexican immigrants (n = 140) reported that they were satisfied with life, with 52% reporting being “somewhat happy” and 40% reporting being “extremely happy,” and only 7% reporting being “generally disappointed” [24]. Mexican immigrants in this sample reported that the US is better than their host country at offering the opportunity to earn a good living, have a trustworthy legal system, provide good health care, and maintain a good education system [24]. Another study compared life satisfaction across race/ethnicity in the US, and contrary to their hypothesis, life satisfaction was not lower among Mexican-origin Latinos as compared to other Latinos [25]. In a sample of middle-aged Mexican Americans living in the Southwestern US, life satisfaction scores were high (M = 40, SD = 6.58; Range: 10–50; [26]). In this sample, familism and being bilingual were associated with higher levels of life satisfaction.

Mexican immigrants often migrate in hopes of a better life not only for themselves but also for their families. Studies, however, have not considered how life satisfaction changes among immigrants and US-born individuals of Mexican descent living in the US. Research comparing life satisfaction across race/ethnicity in the US has called for the incorporation of the nativity status of Latinos and has noted several psychosocial stressors related to nativity status that may impact life satisfaction [18]. Stress from social isolation, for example, may be particularly salient for recent immigrants and negatively impact life satisfaction. On the other hand, protective factors such as strong family and community ties among immigrants may help bolster life satisfaction. Also, research has found significant relationships between nativity status and discrimination, whereby greater discrimination is reported by individuals born in the US [27]. More experiences with discrimination among Mexicans born in the US may result in lower reports of life satisfaction as compared to the foreign-born [20,28].

### 1.2. Quality of Life

In addition to life satisfaction, the present study also examines overall quality of life, as well as two important quality-of-life-related indicators among Latinos, perceived social support and mental distress. Quality of life is a distinct concept, but related to life satisfaction [29]. Quality of life has been defined as an individual’s perception of his or her mental and physical health and can be operationalized as overall self-rated health [29]. Because life satisfaction is considered a broader construct than self-rated health, the predominant approach when examining relationships between these two variables is to have life satisfaction as the outcome variable and self-rated health as the independent variable [29]. Studies show that better self-reported health is associated with higher levels of life satisfaction, and there is evidence that health is one of the strongest predictors of life satisfaction [29,30]. This research, however, has been largely conducted outside of the US. Few studies have examined the relationship between life satisfaction and quality of life in the US, and studies have typically focused on the general US population [31,32]. Research is needed that examines this relationship among Latinos of Mexican descent living in the US as they make up the majority of the Latino population, particularly in a state like California. Studies suggest that life satisfaction is mediated by cultural values [32]. Research that examines the relationship between life satisfaction and quality of life in the general US population precludes an understanding of the unique experiences of Mexicans/Mexican Americans living in the US. Experiences of immigration, for example, may impact perceptions of quality of life that, in turn, affect life satisfaction. Mexican immigrants may rate their quality of life and life satisfaction high because they have more economic opportunity in the US, or perceive more opportunity for their children, as compared to their home country. Also, for immigrants who migrated as young adults and stayed in the United States most of their adult life the process of downward and social comparison may account for their high levels of happiness as they compare themselves to those left behind [20,33,34]. First-generation immigrants who come over as young adults tend to maintain strong ties with their home countries [35]. Alternatively, Mexicans born in the US, who are of disproportionately low socioeconomic status, may consider their quality of life and life satisfaction low as compared to others in the US [9].

Social support via extended family networks is an important component of the quality of life among Latinos, in particular for those of Mexican descent [36]. The immigrant social ties hypothesis suggests that social ties weaken among Latino immigrants as they become more exposed to cultural norms in the US, and this phenomenon is deleterious to one’s well-being [36]. Findings from studies that examined the relationship between social support and immigration status among Latinos are mixed. Some studies have found that Latino immigrants have larger social networks as compared to later generations, whereas others find the opposite [36]. Additional research is needed to examine the immigrant social ties hypothesis among Latinos of Mexican descent to clarify previous inconsistent findings.

Mental health, another component of quality of life, has been related to life satisfaction, and differences in mental health have been found across nativity status. Among Latinos of Mexican descent, those born in the US report higher rates for many psychiatric disorders as compared to immigrants [37,38]. Research examining mental distress, as opposed to psychiatric disorders, by nativity status is more limited. Mental distress is an important construct to examine as it is more common than psychiatric disorders. Furthermore, mental distress among Latinos may be on the rise as more exclusionary immigrant policy climates develop in the US [8].

Thus, the present study examined how nativity status and quality of life indicators are related to life satisfaction using a large California population-based survey of foreign-born and US-born Latinos of Mexican descent. Overall, studies suggest that Latino immigrants are optimistic about life in the US, both for themselves and their children [1,2,24]. The present study examines whether these expectations are met. The study had two major aims. The first aim was to examine life satisfaction by nativity status, specifically comparing reports of life satisfaction between Mexican immigrants and Mexican Americans born in the US. Mexican Americans born in the US were further divided into two groups: (1) participants who were born in the US, but whose parents were born outside of the US (first-generation), and (2) participants who were born in the US and whose parents were born in the US (second-generation and higher). The second aim was to examine how nativity status and quality of life indicators are related to life satisfaction. Due to limited research examining these relationships, an exploratory approach was taken.

## 2. Materials and Methods

### 2.1. Data Sources

Data for the present study came from the California Quality of Life Surveys (CAL-QOL II and Cal-QOL III) [39,40,41,42]. Both are population-based mental health surveys of the noninstitutionalized California population. Their primary goal was to identify sexual orientation-linked differences in quality of life and mental health measures; we use them here to take advantage of their extensive information on Mexican Americans living in California. Each Cal-QOL was a cross-sectional follow-back survey of respondents previously interviewed in two waves of the California Health Interview Survey (CHIS; [43,44]). Across these two CHIS surveys, more than 90,000 Californians, age 18 and older, were interviewed via random digit dialing (RDD) methods, for the most part in English or Spanish (98% of CHIS respondents). During the health interview, participants were asked for permission to be recontacted for additional studies and those between 18 and 70 years of age were queried for their sexual orientation identity. From the original CHIS sample, 56,621 individuals met eligibility requirements for Cal-QOL participation (i.e., between 18 and 70 years of age at time of CHIS interview, agreed to be recontacted, asked about their sexual orientation, and interviewed in either English or Spanish). Of these, 9238 individuals were selected for possible participation in the Cal-QOLs using stratified methods. In one stratum, all who self-reported a sexual minority orientation were sampled with certainty (n = 2562). In a second, 6676 of the remaining eligible participants were selected proportional to their representation in the California population. The Cal-QOL then attempted to re-interview eligible individuals. Of the 5000 potentially eligible Cal-QOL II participants, 2815 (57%) were successfully interviewed; of the 4238 potentially eligible Cal-QOL III participants, 2449 (58%) were interviewed. In both Cal-QOLs, respondents were administered a nearly identical, fully structured computer-assisted telephone interview (CATI) by extensively trained interviewers. In the combined datasets (n = 5264) 893 respondents reported being of Mexican ethnic origin. These individuals comprise the current study sample. Many (41%) were interviewed in Spanish, including 75% of individuals who reported foreign birth. Approximately two-thirds of foreign-born individuals reported being US citizens and 84% had lived in the US for more than 10 years. Informed consent was obtained from all individual participants included in the study. This resulted in three groups of Mexican descent participating in our study. Study participants were classified as foreign-born if they indicated that they themselves immigrated here to the United States (n = 460) The second group was classified as US-born if they indicated that at least one of their parents was foreign-born and the study participant was born in the United States (n = 248). The third group is US-born with Mexican descent parents who were also born in the US (n = 185).

### 2.2. Measures

The Cal-QOL interview assessed both physical and mental health status using the 4-item Healthy Days Core Module (HRQOL–4; [44,45]), other quality of life indicators, life satisfaction, and demographic information. For the current study, we included the following measures:

#### 2.2.1. Frequent Mental Distress

In the HRQOL-4, respondents were asked “Now thinking about your mental health, which includes stress, depression, and problems with emotions, for how many days during the past 30 days was your mental health not good?”. Responses ranged from 0–30 days. Individuals reporting 14 or more days of poor mental health in the past month we classified as experiencing frequent mental distress [44].

#### 2.2.2. General Health Status

Also, in the HRQOL-4, respondents were asked to rate their general health on a 5-point scale (“Would you say that in general your health is excellent, very good, good, fair, or poor?).

#### 2.2.3. Social Support

Social support was measured using a 6-item scale drawn from the Health Care for Communities Survey [44]. Specifically, respondents were asked about the availability of social support resources in the 4 weeks prior to interview. Dimensions assessed included emotional support (“someone to love and make you feel wanted,” “someone to confide in or talk to about yourself or your problems”), tangible support (“someone to help with daily chores if you were sick,” “someone to give you money if you needed it”), informational support (“someone to give you information to help you understand a situation”), and companionship (“someone to have a good time with”). Answer options included “none of the time,” “a little of the time,” “some of the time,” “most of the time,” and “all of the time.” Items were summed to create a social support score with values ranging from 0 to 24 [46]. For the current study, the items were highly intercorrelated (Cronbach’s Alpha = 0.83).

#### 2.2.4. Life Satisfaction

Life satisfaction was measured using 8 items drawn from the Australian Unity Well-being Index [47]. One item assessed overall life satisfaction: “All things considered, how satisfied would you say you are with your life as a whole? Would you say very satisfied, satisfied, neither satisfied nor dissatisfied, dissatisfied, or dissatisfied?” Seven items assessed satisfaction in specific domains (standard of living, health, life achievements, personal relationships, safety, community connections, and future safety). Specifically, respondents were asked “How satisfied are you with… (your standard of living, your health, what you are achieving in life, your personal relationships, how safe you feel, feeling part of your community, your future security)?” Responses for each of the items ranged from 0 (“very dissatisfied”) to 4 (“very satisfied”). These items were summed to create a Personal Well-Being Index (PWI). Scoring for the PWI is standardized to a 0-to-100-point scale with 100 indicating complete satisfaction with life. As expected, items were highly intercorrelated (Cronbach’s Alpha = 0.86) suggesting excellent internal reliability in measuring life satisfaction. 

#### 2.2.5. Individual Characteristics

Finally, the Cal-QOL also ascertained several demographic characteristics including gender, age, racial/ethnic background, foreign birth, years in the US if foreign-born, educational attainment, relationship status, sexual orientation, household composition, languages spoken within the household, family income, and parental nativity. From this, we classified respondents for generational status: foreign-born (n = 460), US-born but having one or more parent who was foreign-born (n = 248), and US-born with parents also US-born (n = 185). We also created the following variables: gender (male, female), age (in years), sexual orientation (lesbian, gay, bisexual vs. heterosexual), educational attainment (less than high school, high school degree, some college, college degree or more), relationship status (married or cohabiting vs. not), presence of children under age 18 living in the household (yes, no), family income less than 200% of the federal poverty level (yes, no), and Spanish spoken within the household (yes, no).

### 2.3. Data Analytic Plan

Data were analyzed using SUDAAN 11.0 [48]. All analyses were weighted to adjust for selection probability, nonresponse, and post-stratification to the California population. In the first set of analyses, we examined demographic differences among individuals of different generational statuses. To do so, we used a multinomial logistic regression equation regressing generational status on all demographic variables and survey cycles simultaneously. Next, we investigated generational differences in quality-of-life indicators and health status using either Wald Χ2 tests for categorical outcomes or Wald F tests for interval-scaled outcomes. For the latter, we conducted post-hoc comparisons of foreign-born Mexican Americans to U.S.-born Mexican Americans with any foreign-born and U.S.-born parents. Finally, we used multiple regression methods to predict life satisfaction in a sequential set of models. Model 1 sought to predict life satisfaction including generational status and ascribed characteristics (e.g., age, gender, sexual orientation) as predictors. Model 2 added achieved characteristics (e.g., educational attainment, relationship status, family income, presence of children, and Spanish spoken within the home). Model 3 added quality of life indicators (e.g., social support, frequent mental distress, and self-rated health). Analyses were also adjusted for survey cycle. We report point estimates and their standard errors (S.E.), results of Wald tests, and estimates of slope (betas) from the regression analyses and their S.E. We also report marginal means and their 95% confidence intervals for life satisfaction after adjusting for ascribed, achieved, and quality of life indicators. Statistical significance was evaluated at the *p* < 0.05 level.

## 3. Results

### 3.1. Characteristics of the Sample

Mexican American Cal-QOL respondents varied somewhat in their demographic characteristics by generational status (See Table 1). While we observed no evidence of statistically significant differences in gender, sexual orientation, or presence of children in the household, foreign-born individuals were somewhat older (Wald F (2) = 6.80, *p* < 0.01), reported lower levels of educational attainment (Wald F (6) = 6.65, *p* < 0.001), were more likely to be married or living with a relationship partner (Wald F (2) = 7.29, *p* < 0.01), had lower family income (Wald F (2) = 4.27, *p* < 0.05), and were more likely to report speaking Spanish at home (Wald F (2) = 59.10, *p* < 0.001).

### 3.2. Quality of Life Indicators

Generational status was associated with perceptions of social support (Wald F (2) = 23.51, *p* < 0.001) (see Table 2). In post-hoc comparisons, foreign-born Mexican Americans reported lower levels of social support than US-born Mexican Americans with either at least one foreign-born parent (Wald F (2) = 26.70, *p* < 0.001) or only US-born parents (Wald F (2) = 36.53, *p* < 0.001). Self-rated health also varied (Wald X2 (8) = 8.80, *p* < 0.001) with foreign-born individuals reporting somewhat worse health. However, there was no difference detectable in the prevalence of frequent mental distress.

### 3.3. Predictors of Life Satisfaction

Life satisfaction scores did not differ significantly among individuals of different generational statuses in the absence of controlling for confounding. In Table 3, we report the results of three sequential multiple regression models regressing life satisfaction on generational status while controlling for differences in individual backgrounds, socioeconomic and family life characteristics, and quality of life indicators (see Table 3). In the initial model predicting life satisfaction from generational status, age, gender, and sexual orientation, only the female gender (Wald F (1) = 4.51, *p* < 0.05) predicted higher levels of life satisfaction. In Model 2, we added to the original model socioeconomic and family life characteristics (i.e., educational attainment, relationship status, family income, presence of children in the household, and speaking Spanish at home). Here again, we observed that women report greater life satisfaction than men (Wald F (1) = 6.22, *p* < 0.05), as do those with higher levels of education (Wald F (3) = 5.87, *p* < 0.001), higher family income (Wald F (1) = 12.53, *p* < 0.001), and partnered individuals (Wald F (1) = 8.47, *p* < 0.01). Notably, the foreign-born were more satisfied than first-generation US-born Mexican Americans (Wald F (1) = 5.64, *p* < 0.05). In the third model, we added quality of life indicators (i.e., social support, mental distress, and self-rated health). Here, too, higher levels of life satisfaction were associated with being female (Wald F (1) = 7.05, *p* < 0.01), as well as reporting more social support (Wald F (1) = 40.20, *p* < 0.001), an absence of frequent distress (Wald F (1) = 41.46, *p* < 0.001), and better physical health (Wald F (1) = 15.28, *p* < 0.001). With this confounding adjustment, generational status was strongly associated with life satisfaction such that the foreign-born were more satisfied than first-generation US-born Mexican Americans (Wald F (1) = 18.70, *p* < 0.001) and US-born Mexican Americans whose parents were also US-born (Wald F (1) = 12.09, *p* < 0.001). After adjusting for confounding, estimated life satisfaction mean scores for foreign-born Mexican Americans ($\bar{X}$ = 78.2, 95% CI: 76.9–79.6) greatly exceeded that of US-born Mexican Americans with at least one foreign-born parent ($\bar{X}$ = 72.2, 95% CI: 70.0–74.4) or only US-born parents ($\bar{X}$ = 72.5, 95% CI: 69.8–75.2). Average levels of life satisfaction among US-born Mexican Americans did not differ by generational status.

## 4. Discussion

The key aim of this study was to explore the role of Mexican-origin nativity in life satisfaction; that is, how do foreign-born Mexicans living in the US differ from US-born Mexican Americans in their sense of life satisfaction? On the concept of life satisfaction, the data are clear—US-born Mexican Americans have lower life satisfaction as compared to their Mexican immigrant counterparts [20,28]. There were no differences in life satisfaction, however, across first and second-generation and higher US-born groups. In the present study, life satisfaction among the foreign-born was high, consistent with previous research showing high rates of life satisfaction among Mexican immigrants living in the US [24]. Within the context of immigration, the American dream suggests that life satisfaction should improve for the children of Mexican immigrants through hard work and the rewards associated with hard work [7,49,50]. However, findings from this study indicate that life satisfaction was lower among the US-born. 

Sociodemographic characteristics associated with life satisfaction included gender, education, family income, and marital status. Although the US-born were less likely to be married or cohabiting, they generally had more education and higher income than their foreign-born counterparts, yet they still reported lower life satisfaction. There were no differences across nativity status groups by gender. Although the US-born had achieved more than the immigrant group in terms of education and income, these achievements did not translate to satisfaction with life. Second-generation and higher Mexican Americans were similar to the first-generation according to most sociodemographic characteristics, except they were somewhat more acculturated, judging by the proportion that speak Spanish at home [50,51]. More first-generation Mexican Americans likely spoke Spanish at home to communicate with their parents who are likely to be monolingual or more comfortable communicating in Spanish. The similarities between the foreign-born who immigrated and those who had a foreign-born parent but were born in the United States and higher groups suggest less social mobility occurred between these groups, and also little differences in life satisfaction.

Central to the broader concept of life satisfaction is the construct of quality of life, which is often equated with social support and mental and physical health. In this study, the level of mental distress was not significantly different across nativity status; however, the US-born reported more perceived social support and better overall health than the foreign-born. Even though the data show that US-born Mexican Americans have a higher quality of life according to these indicators, this did not translate into a sense of life satisfaction. Interestingly, although immigrants are found to have strong social ties with friends, family, and community that act as protective factors in dealing with negative outside forces, US-born Mexican Americans perceived their social support as stronger than that of the foreign-born [36]. Perhaps social ties or social support are culturally based. When one lives in an ethnic enclave, as is common among the foreign-born, there is often a high degree of interaction, but these ties may be viewed as natural and not necessarily as a “support system” [36,51]. Additionally, la familia is regarded as the major source of support so that outside members—friends and community members—may not be viewed as the core support system [35]. Marriage or having a co-habiting partner is also generally viewed as a strong source of social support; however, the US-born Mexican sample was almost half as likely to be married or co-habiting compared to the foreign-born, resulting in differential access to traditional sources of social support. US-born Mexican Americans may have larger social support networks because they are more likely to include friends in their support system and not just family members, yet these support networks did not translate into improved life satisfaction. It may be that these support networks of friends serve as a frequent source of comparison of the American dream. As compared to others in the US, US-born Latinos reach lower levels of attainment of the American dream in that they earn less, receive fewer higher education degrees, and overall work at jobs with less prestige and economic benefits compared to other groups in society, potentially accounting for their lower levels of life satisfaction [52,53,54]. 

In addition, although self-rated health scores were higher among the US-born group as compared to the foreign-born, once again, this did not translate to more satisfaction with life. Our findings suggest that there may be other factors operating for US-born individuals of Mexican origin living in the US, besides social support and self-rated health, that play an influential role in shaping life satisfaction. 

Two potential factors may contribute to our understanding of this difference in perceived life satisfaction between the US and foreign-born: (1) the standard of comparison for life satisfaction, and (2) discrimination. Life satisfaction can be defined as achieving a position in society that one expects to be correlated with hard work. Namely, the American dream, where if you work hard opportunities will occur that will allow you to achieve in society. Theoretical discussions of life satisfaction note the importance of the standard referent to which one’s life is being compared, and discuss the subjective nature of the referent [13]. For Mexican immigrants, the standard referent for life satisfaction may be based on the country from which the person immigrated or to other individuals in social networks who also immigrated to the US. For immigrants who perceived their past life as lacking economic opportunity to support their families, an educational system to educate their children, and a health care system to fall back on, their life in the US may be perceived in a positive way, despite significant obstacles to obtain a job, education, or health care. On the other hand, the referent for US-born Mexican Americans may be other individuals in the US, not just other Latinos. Overall, Mexican Americans have lower levels of education and income as compared to the general US population [51]. US-born Mexican Americans who compare themselves to others in the US may be more likely to view their current life circumstances as below their standard referent and as not having progressed as far as the dream they or their parents had in mind. Other studies have noted a similar finding indicating that even in US-born Latinos with education they are less satisfied with their life in comparison to Whites [28,55,56]. The speculation is that this stemmed from the discrepancy in their expectations for achievement. Calvo and colleagues (2017) in their study noted that US-born Latinos reported more daily experiences of discrimination than any other racial/ethnic or White group even more than immigrant Latinos [28]. They offer the hypothesis that the life dissatisfaction of the US-born educated immigrant may be shaped by their experiences of structural discrimination that impede socioeconomic advancement. 

While such discrimination was not the focus of our study indeed racial/ethnic and socioeconomic discrimination may impact life satisfaction and account for the differences found across nativity status. US-born Mexican Americans often face discrimination socially and economically [54]. Many US-born Mexican Americans feel unaccepted in certain social circles and in fact, are excluded in many [54]. They are one of the groups addressed by affirmative action policy, which for some suggests a class that needs to be given opportunity [5]. In addition, they may suffer discrimination that targets Mexican immigrants even though they are US-born. They are underrepresented in middle and upper socioeconomic status levels of society and continue to be overrepresented among the poor, despite being born in the US [52]. When they view others, who have achieved more than they have, given similar attributes but belonging to other racial/ethnic groups, they may experience the American Dream as not working as equally well for them [54]. Furthermore, research among Mexican Americans shows greater experiences of discrimination among individuals who have more contact with non-Hispanic Whites [57]. In the present study, the US-born sample with US-born parents was more likely to speak English at home, suggesting higher levels of acculturation in this group [50]. Higher levels of acculturation may result in greater contact beyond just with other Latinos exposing them to greater discrimination.

There are limitations to the present study. Participants in this study were drawn only from California and were all of Mexican origin, limiting the generalizability of findings to other areas in the US and to Latinos of different ethnic backgrounds. In addition, data on the length of time participants and their parents lived in the US was not available for the US-born, limiting further exploration of acculturation issues that have been found to impact well-being and quality of life among Latinos. Finally, the present study was cross-sectional, precluding discussion of causality.

## 5. Conclusions

Despite the aforementioned limitations, the clarity of our findings about the decreasing sense of well-being as Mexican Americans become more distal to the immigration process raises concerns. Findings from this study alert us to potential changes in the well-being of US-born Latinos of Mexican descent. Given the changing demographic landscape in the US with more native births driving Mexican American population growth as compared to immigration, as this population grows to be a majority in some parts of the US, it is important that we address this lack of life satisfaction and well-being. US-born Latinos of Mexican descent are not finding access to opportunities like their peers, and the American dream that previous immigrant generations sacrificed for is being seen as less of a reality among US-born Latinos of Mexican descent. As compared to non-Hispanic Whites, Mexican immigrants and their children can experience difficulty in obtaining opportunities in the US that were their dream: education, fair wage employment, income, and jobs/careers [58,59]. 

Studies that can identify mediators of the relationship between nativity, quality of life indicators, and life satisfaction may provide important insights for interventions that can increase well-being and protect against the negative physical and mental health outcomes associated with lower levels of life satisfaction. Mexican Americans, particularly in states like California and Texas, are destined to become the majority. As we worry about the cost of health care, one step towards such cost containment is to address the life satisfaction and well-being of Mexican Americans. This can occur through addressing equity in access to education, employment, and income in order to facilitate their achievement of the American dream. In the short term, gaining a better understanding of the mediators of life satisfaction in US-born Latinos of Mexican descent as well as examining whether the increase in anti-immigration sentiment is affecting their sense of capabilities to achieve the American dream and contribute to society are important to addressing well-being in their lives.

## Figures and Tables

**Table 1:** Demographic characteristics of Mexican Americans, aged 18 to 72 years, by nativity and generational status in the California Quality of Life Surveys. N = 893. Weighted estimates (percentages or means) and standard errors shown. Differences in demographic characteristics evaluated by a single multinomial logistic regression in which generational status was regressed on all predictors and survey cycles simultaneously. SE = Standard error; Est = Estimate.

	Foreign Born (n = 460)		US-Born/ At Least One Foreign-Born Parent (n = 248)		US-Born/ No Foreign-Born Parent (n = 185)	
Characteristics	Est.	(SE)	Est.	(SE)	Est.	(SE)
Female gender, %	53.5	(2.6)	50.4	(3.7)	51.1	(4.6)
Age, $\bar{X}$ years **	40.2	(0.6)	32.7	(1.0)	38.0	(1.4)
Lesbian, gay, or bisexual, %	1.3	(0.3)	2.7	(0.6)	3.9	(0.8)
Education, % ***						
Less than High School	48.7	(2.6)	9.8	(2.1)	9.8	(2.6)
High School degree	27.6	(2.4)	34.2	(3.6)	31.1	(4.1)
Some college	15.1	(1.9)	33.2	(3.6)	33.8	(4.4)
College degree or more	8.5	(1.5)	22.8	(3.0)	25.3	(4.1)
Married or cohabiting, % *	72.8	(2.4)	43.2	(3.7)	48.6	(4.6)
Children in household, %	68.1	(2.5)	53.6	(3.7)	40.5	(4.5)
Family income < 200% of Federal poverty level, % *	60.3	(2.6)	38.6	(3.6)	32.4	(4.2)
Spanish spoken in home, % ***	94.9	(1.2)	81.8	(2.7)	29.1	(4.1)

* *p* < 0.05. ** *p* < 0.01. *** *p* < 0.001.

**Table 2:** Quality of life indicators and life satisfaction among Mexican Americans, age 18 to 72 years, by immigrant generational status in the California Quality of Life Surveys. N = 893. Weighted estimates and standard errors shown. Prevalence sums to 100%, except for rounding error. Differences evaluated by Wald χ2 tests for categorical variables and Wald F-test for interval-scaled variables. Est. = estimate; SE = Standard error.

	Foreign Born (n = 460)		US-Born/ At Least One Foreign-Born Parent (n = 248)		US-Born/ No Foreign-Born Parent (n = 185)	
Characteristics	Est.	(SE)	Est.	(SE)	Est.	(SE)
Quality of life indicators						
Perceived social support, $\bar{X}$ (SE) ***	16.0	(0.3)	18.5	(0.4)	19.3	(0.5)
Frequent mental distress, % (SE)	11.9	(1.7)	8.9	(2.2)	10.0	(2.6)
Self-rated health, % (SE) ***						
Excellent	13.1	(1.8)	18.1	(2.9)	12.4	(3.0)
Very good	16.1	(1.9)	29.2	(3.4)	45.0	(4.6)
Good	39.0	(2.6)	41.8	(3.7)	26.1	(4.0)
Fair	26.6	(2.4)	8.6	(2.0)	10.5	(2.7)
Poor	5.3	(1.2)	2.2	(1.1)	6.0	(2.1)
Life satisfaction, $\bar{X}$ (SE)	76.5	(0.8)	74.2	(1.3)	74.9	(1.5)

*** *p* < 0.001.

**Table 3:** Predictors of life satisfaction among Mexican Americans, age 18 to 72 years, in the California Quality of Life Surveys. N = 893. Models estimated by multiple regression. *b* = beta; SE = Standard error.

	Model 1: Generational Status and Ascribed Controls		Model 2: Model 1 Plus Achieved Controls		Model 3: Model 2 Plus Quality of Life Indicators	
Predictor	*b*	(SE)	*b*	(SE)	*b*	(SE)
Generational status (ref = foreign-born)						
US-born—parents foreign-born	−2.12	(1.54)	−3.82	(1.61) *	−6.07	(1.40) ***
US-born—parents US-born	−1.22	(1.73)	−2.63	(2.01)	−5.74	(1.65) ***
Age	−0.01	(0.05)	−0.04	(0.05)	0.07	(0.04)
Female gender	2.66	(1.25) *	3.08	(1.24) *	2.70	(1.02) **
Lesbian, gay, bisexual identity	−4.52	(2.54)	−2.95	(2.37)	−2.82	(1.99)
Educational attainment (ref = less than HS)						
High school degree			2.55	(1.58)	−0.34	(1.39)
Some college			5.79	(1.95) **	1.67	(1.66)
College degree or more			7.58	(1.91) ***	1.61	(1.74)
Married or cohabiting			5.11	(1.44) ***	1.00	(1.27)
Family income less than 200% of FPL			−4.19	(1.44) **	−1.44	(1.24)
Children live in household			0.71	(1.41)	0.92	(1.17)
Spanish spoken at home			1.45	(1.69)	0.63	(1.40)
Perceived social support					0.77	(0.12) ***
Frequent mental distress					−13.17	(2.05) ***
Self-rated health (ref = poor health)						
Fair					8.53	(2.73) ***
Good					10.60	(2.61) ***
Very good					15.92	(2.86) ***
Excellent					19.51	(2.94) ***
Survey cycle (ref = Cal-QOL II)	−1.41	(1.27)	−0.76	(1.27)	−1.03	(1.05)
Constant	76.12	(2.29) ***	71.71	(3.38) ***	49.16	(4.05) ***
R^2^	0.02		0.09		0.35	

* *p* < 0.05. ** *p* < 0.01. *** *p* < 0.001.

## Data Availability

The California Quality of Life Survey is not a public dataset at this time. Those interested in data access can contact Susan D. Cochran (cochran@ucla.edu) of the following studies: https://doi.org/10.1037/ccp0000047 and https://doi.org/10.15288/jsad.2012.73.675. The California Health Interview Surveys from different years can be found here: 5 January 2022 https://healthpolicy.ucla.edu/chis/data/Pages/GetCHISData.aspx.
